# Chromosomal over-replication in *Escherichia coli recG* cells is triggered by replication fork fusion and amplified if replichore symmetry is disturbed

**DOI:** 10.1093/nar/gky566

**Published:** 2018-06-30

**Authors:** Sarah L Midgley-Smith, Juachi U Dimude, Toni Taylor, Nicole M Forrester, Amy L Upton, Robert G Lloyd, Christian J Rudolph

**Affiliations:** 1 Division of Biosciences, College of Health and Life Sciences, Brunel University London, Uxbridge UB8 3PH, UK; 2 Medical School, Queen's Medical Centre, Nottingham University, Nottingham NG7 2UH, UK

## Abstract

Chromosome duplication initiates via the assembly of replication forks at defined origins. Forks proceed in opposite directions until they fuse with a converging fork. Recent work highlights that fork fusions are highly choreographed both in pro- and eukaryotic cells. The circular *Escherichia coli* chromosome is replicated from a single origin (*oriC*), and a single fork fusion takes place in a specialised termination area opposite *oriC* that establishes a fork trap mediated by Tus protein bound at *ter* sequences that allows forks to enter but not leave. Here we further define the molecular details of fork fusions and the role of RecG helicase in replication termination. Our data support the idea that fork fusions have the potential to trigger local re-replication of the already replicated DNA. In *ΔrecG* cells this potential is realised in a substantial fraction of cells and is dramatically elevated when one fork is trapped for some time before the converging fork arrives. They also support the idea that the termination area evolved to contain such over-replication and we propose that the stable arrest of replication forks at *ter*/Tus complexes is an important feature that limits the likelihood of problems arising as replication terminates.

## INTRODUCTION

**Figure 1.**
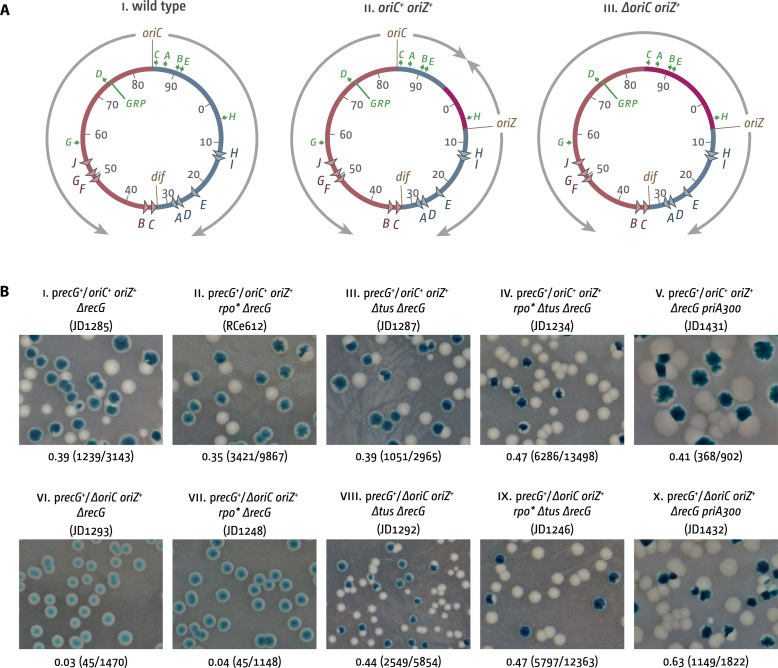
DNA replication dynamics in cells with a single replication origin either in its native or in an ectopic location as well as in cells with two replication origins. (**A**) Schematic representation of the replichore arrangement of one or two replication origins in *E. coli*. The origins *oriC* and *oriZ* as well as the *dif* chromosome dimer resolution site are highlighted. Replichores and replication directionality are indicated by grey arrows. *ter* sites are indicated by triangles and identified by their corresponding letter (‘A’ indicates the *terA* site). The numbers represent the minutes of the standard genetic map (0–100 min). Green arrows represent location and direction of transcription of the 7 *rrn* operons *A–E, G* and *H*. The location marked ‘GRP’ indicates a tight cluster of genes coding for ribosomal proteins, all of which are transcribed co-directionally to replication coming from *oriC*. Chromosomal sections in which the direction of DNA replication is artificially inverted because of the presence of an ectopic replication origin are shown in purple. (**B**) Maintenance of viability of *oriC^+^ oriZ^+^* and *ΔoriC oriZ^+^* cells in the absence of RecG. The plate photographs shown are of synthetic lethality assays, as described in Materials and Methods. The relevant genotype of the construct used is shown above each photograph, with the strain number in parentheses. The fraction of white colonies is shown below, with the number of white colonies/total colonies analysed in parentheses. The plasmid used was pJJ100 (*recG^+^*) (see Supplementary Information).

Every time a cell divides, its DNA content has to be replicated and transmitted to its daughter cells with high fidelity (1). Failure to do so can be fatal, or lead to mutation and genomic instability, the root causes of cancer. Replication of the single circular chromosome of *Escherichia coli* initiates at a single origin (*oriC*) via the action of the initiator protein DnaA, which facilitates assembly of two replication fork complexes (replisomes) that move away from the origin in opposite directions, replicating the DNA at rates of 650–1000 nt/s (2). Replication is completed when converging forks fuse within a specialized termination region flanked by polar *ter* sequences (*terA–J*) that are bound by the Tus terminator protein (Figure 1A) (3,4). This region also contains specialized genetic elements such as the *dif* site that facilitates resolution of any chromosome dimers and KOPS sequences which guide proteins facilitating the segregation of duplicated DNA to daughter cells (5,6). The *ter* sites are oriented such that when bound by Tus they form a strong replication fork pause site (3,7). The presence of multiple *ter* sites generates a termination area that allows forks to enter but not to leave (Figure 1A) (3,7–8). Thus, the chromosome is divided into two approximately equal halves called replichores, each replicated by a single replication fork complex (9).

While initiation and elongation of DNA replication, as well as many of the final steps of chromosome duplication such as decatenation and resolution of chromosome dimers, are generally well understood in *E. coli*, the events associated with fusion of two replisomes are only beginning to emerge. Recent studies suggest that RecG protein, a multifunctional DNA translocase that remodels a variety of branched DNA structures *in vitro* (10–12), plays a significant role at this stage. Marker frequency analyses of exponentially growing cells lacking RecG have revealed substantial over-replication of sequences in the termination region, indicating that events associated with the termination of replication have the potential to trigger aberrant DNA synthesis, and that RecG normally curbs such events. We have suggested previously that during the fusion of two replication forks the DnaB helicase of one fork sometimes might displace the leading strand of the opposing fork, resulting in the formation of a 3′ ssDNA flap structure (13–15) (see also Figure 7). Displacement of a 3′ flap is not observed with DnaB alone (16), but over-replication of the leading strand is observed *in vitro* with an *oriC* plasmid template and reconstituted replisomes (17), showing that nascent leading strand displacement is a particular risk following collision of two replisomes (12,15,18). Normally, these flaps would be eliminated by a 3′ single-stranded DNA exonuclease, or converted to a 5′ flap structure by RecG helicase. RecG has the necessary activity to unwind the 5′ end at the branch point of a 3′ flap while simultaneously reannealing the 3′ single-strand flap (10,19–21). The resulting 5′ flap could then be removed by a 5′ single-stranded DNA exonuclease. In the absence of RecG, 3′ flaps persist for longer and are targeted by the primosome assembly factor PriA to establish new replication forks that re-replicate the termination area via the establishment of D-loop recombination intermediates. The model proposed is able to explain that (a) cells lacking the major 3′ exonucleases show similar over-replication in the terminus area (13), (b) RecG is needed to keep these exonuclease-deficient cells alive (22) and (c) over-replication in cells lacking RecG requires the helicase activity of PriA and, more specifically, its ability to unwind 3′ flaps (13,14).

RecG is a double-stranded DNA translocase that unwinds a variety of branched substrates *in vitro* (10–12). It is therefore no surprise that several explanations for the origin-independent replication in *ΔrecG* cells have been discussed (23–27). RecG has been shown to unwind R-loops and D-loops *in vitro* (12,26) and cells lacking RecG exhibit origin-independent DNA synthesis, which was thought to initiate at persistent R-loops, similar to the origin-independent synthesis in cells lacking RNase HI (28). It was therefore suggested that the origin-independent synthesis in *ΔrecG* cells might be triggered by increased levels of D- or R-loops specifically arising within the termination area (24,27). Yet further possible explanations follow from the recent discovery that over-replication of the terminus area occurs not only in cells lacking RecG or 3′ single-strand DNA exonucleases, but also in cells lacking DNA polymerase I (18) or the RecD component of RecBCD enzyme necessary for its ATP-dependent exonuclease activity (25,29). Azeroglu and colleagues proposed that RecG might be involved in the reverse-restart of arrested replication forks, triggering over-replication in the termination area as forks get arrested at *ter*/Tus complexes (23,30). Wendel *et al*. proposed that replisomes might move past each other, thereby over-replicating a stretch of the chromosome (25,29), an idea similar to that observed during termination in eukaryotic cells (31). It was also suggested that torsional stress between two merging forks might force both to reverse (27). Most of these models invoke the formation of double-stranded DNA ends that are processed by the RecBCD and RecA recombinases, leading to the formation of D-loops that prime further replication (25,27,30).

In this study, we have further defined the mechanics of termination and the role that RecG plays in preventing over-replication of the terminus area. We show that the broadly-defined over-replication observed in an ectopic termination area is converted to a well-defined peak if the region where most forks meet is flanked by *ter*/Tus complexes. These and other data support the idea that the over-replication seen in the absence of RecG arises largely from pathological events associated with the head-on fusion of fork complexes. Importantly, it does not require forks to be blocked at *ter*/Tus complexes, but appears to be exacerbated when one fork is trapped for some time before the converging fork arrives. The data also indicate that the formation of aberrant fork structures during the termination of replication is a general feature of the cell population and can lead to cell inviability if exacerbated, especially if repair mechanisms are not in place to deal with the consequences. Our data strongly support the idea that the termination area has evolved to contain any re-replication triggered, as suggested (3–4,13,18,32). They also indicate that maintaining the integrity of a replisome complex following its arrest at *ter*/Tus is not simply a manifestation of the mechanics of arrest but a key feature that reduces the likelihood of re-replication as forks merge and fuse. We propose that the stable arrest of one fork before the converging fork arrives helps to avoid the generation of pathological DNA structures that PriA can target to trigger re-replication.

## MATERIALS AND METHODS

### Bacterial strains and general methods

For *E. coli* K12 strains see Supplementary Table S1. Strains were constructed via P1*vir* transductions (33) or by single-step gene disruptions (34). The *dnaA46* allele encodes a thermosensitive DnaA protein that is inactive at 42°C. For assessing growth without DnaA initiation, cultures of *dnaA46* constructs grown at 30°C to an *A*_600_ of 0.4 were diluted in 10-fold steps from 10^–1^ to 10^–5^ before spotting 10 μl samples of each dilution on LB agar. Duplicate plates were incubated at 30°C and 42°C. To construct strains carrying an ectopic termination area, the *terA* sequence was integrated in two separate chromosomal locations, one at 4.44 Mb in an orientation permissive for a fork coming from *oriC* (*terA4.44*), and the second at 4.57 Mb in an orientation that would block this fork (*terA4.57*) (see schematic in Figure 3A and B). Constructions were carried out in *Δtus* cells to avoid the danger of blocked replication. To test functionality of the ectopic *ter* sites we transferred the *terA4.57* allele in parallel into wild type and *oriC^+^ oriZ^+^* cells (35) via P1*vir* transduction. *terA4.57* was easily transferred into *oriC^+^ oriZ^+^* cells. Viability is expected to be retained in this case, because even if replication forks coming from *oriC* would be blocked, synthesis coming from *oriZ* would complete chromosome duplication. In contrast, we did not observe any transductants when we tried to transduce the *ter4.57* allele into wild type cells, in line with the idea that the ectopic *ter* site blocks synthesis of the clockwise forks, whereas the counter-clockwise fork will get blocked in the native termination area. This suggests that *terA4.57* is functional *in vivo*. We then confirmed via Sanger sequencing that the sequences of both *terA4.44* and *terA4.57* were identical.

### Growth media

Luria broth (LB) and agar was modified from Luria and Burrous (36) as follows: 1% tryptone (Bacto™, BD Biosciences), 0.5% yeast extract (Bacto™, BD Biosciences) and 0.05% NaCl (Sigma Aldrich). The pH was adjusted to 7.4. M9 minimal medium (Bacto™, BD Biosciences) contained 15 g/l KH_2_PO_4_, 64 g/l Na_2_HPO_4_, 2.5 g/l NaCl and 5.0 g/l NH_4_Cl. Before use, MgSO_4_, CaCl_2_ and glucose were added from sterile-filtered stock solutions to final concentrations of 2 mM, 0.1 mM and 0.2%, respectively, according to the manufacturer's recommendation. Doubling times of MG1655 in our growth media were 19.3 ± 1.7 min in LB and 68.8 ± 6.2 min in M9 glucose.

### Synthetic lethality assay

The synthetic lethality assay was performed as described (37,38). In essence, a wild type copy of *recG* under its native promoter was cloned into pRC7, a *lac^+^* mini-F plasmid that is rapidly lost, and used to cover *ΔrecG* in the chromosome, in a *Δlac^–^* background. One or more additional mutations can then be tested for synthetic lethality with the *ΔrecG* allele. If synthetically lethal, cells that lose the plasmid will fail to grow and only *lac^+^* colonies formed by cells retaining the plasmid will be observed. When viability is reduced but not eliminated, the colonies formed by cells retaining the plasmid are noticeably larger than those white colonies formed by plasmid-free cells. To record the phenotype, cultures of strains carrying the relevant pRC7 derivatives were grown overnight in LB broth containing ampicillin to maintain plasmid selection, diluted 100-fold in LB broth and grown without ampicillin selection to an *A*_600_ of 0.4 before spreading dilutions on LB agar or M9 glucose minimal salts agar supplemented with X-gal and IPTG. Plates were photographed and scored after 48 h (LB agar) or 72 h (M9 agar) at 37°C.

### Marker frequency analysis by deep sequencing

Marker frequency analysis by deep sequencing was performed as described (35). See Supplementary Methods for details. All relevant raw sequencing data can be accessed at the European Nucleotide Archive (http://www.ebi.ac.uk/ena/data/view/PRJEB25595).

### Fluorescence microscopy

Cultures of strains carrying the relevant fluorescent protein fusion constructs were incubated in M9 minimal medium with 0.2% glucose until they reached an *A*_600_ of 0.3–0.35. Microscope slides were equipped with a Gene Frame^®^ (ABgene) and filled with M9 minimal medium with 0.2% glucose and 1% agarose. 1.5 μl of the culture was added on top and incubated until all liquid had evaporated, the gene frames sealed with a cover slip and the slides examined using a Nikon T*i*-U inverted microscope equipped with a DS-Qi2 camera (Nikon). YPet fluorescence was visualized using a Nikon yellow fluorescent protein YFP HYQ filter. Images were taken and analysed via Nikon NIS-Elements Br software 4.3 (Nikon) and processed using Nikon NIS-Elements Br and Adobe Photoshop CC. Fluorescent foci were counted visually and only the strain number but not the genotype was known at the time of counting. This blinded analysis ensured that no bias was introduced when foci were counted. The use of YPet-DnaN resulted in clearly defined foci that were easily distinguished from background fluorescence. Very occasionally it was not clear whether cells contained an actual fluorescent focus in a subcellular region or whether background fluorescence was somewhat denser in a small area of the cell. Such cells were rare (<5% of total cells counted) and they were excluded from the analysis.

## RESULTS

A distinct phenotype of the replication profile of logarithmically grown *ΔrecG* cells is a sharply-defined peak of sequence over-replication within the boundaries of the termination area (13–14,29). This feature is much exacerbated in cells with a second ectopic replication origin (*oriZ*) located approximately mid-way along the clockwise replichore (13) (Figure 1A; see also Figure 3C, panel iii). To investigate why over-replication is so pronounced, we attempted to delete *oriC*. However, we found we could do so only provided RecG was expressed in trans from a pRC7 plasmid carrying the wild-type *recG* gene. pRC7 is an unstable plasmid that contains a copy of the *lac* operon. It is rapidly lost if selection is not maintained. In a strain deleted for the chromosomal *lac* operon, the presence or absence of the plasmid can be detected on agar plates containing the β-galactosidase indicator X-gal. A blue colony colour shows the presence of the plasmid (*lac^+^*), while white colonies show the absence of the plasmid. White sectors within blue colonies can be observed if plasmid loss occurs after plating (37,38). This assay revealed that plasmid-free *ΔoriC oriZ^+^ ΔrecG* cells were extremely rare (Figure 1B, panel vi). In addition, the few white colonies observed showed significant size variations indicative of the presence of spontaneous suppressor mutations. Thus, our data strongly suggest that a *ΔoriC oriZ^+^ ΔrecG* strain is inviable. This observation demonstrates that when the chromosome is replicated exclusively from the ectopic replication origin *oriZ*, RecG becomes essential for viability.

Previous studies revealed that deletion of *oriC* from wild type cells carrying an ectopic origin (*oriZ*) compromises viability to some extent, leading to a reduction in growth rate and the accumulation of suppressors that reduce conflicts between transcription and replication (4,35). In the absence of *oriC*, the fork moving counter clockwise from *oriZ* is required to replicate a section of the chromosome containing five highly transcribed *rrn* operons in an orientation opposite to normal and will therefore meet transcription complexes head-on (Figure 1A, panel iii). To investigate whether the dependence on RecG reflects a failure to deal with the aftermath of such collisions, we examined constructs carrying a mutation (rpoB*35) known to destabilise RNAP transcription complexes (35,39–42) and which has been reported to alleviate conflicts between transcription and replication in cells carrying an ectopic replication origin (35). However, we did not see any improvement in viability (Figure 1B, panel vii), indicating that conflicts between replication and transcription are unlikely to be the sole reason for the observed lethality, or if they are then rpo* does not reduce these conflicts to a level permissive for survival.

The initiation of replication at an ectopic origin some distance from *oriC* means that the fork moving clockwise moves past the normal termination area to be held up at *terC* or *terB* for several minutes before the counter clockwise fork arrives. To investigate whether this delay might create a problem only RecG can resolve, we examined the effect of inactivating fork traps by eliminating Tus. Deletion of *tus* indeed suppressed the observed lethality (Figure 1B, panels viii and ix). Thus, RecG is vital in *ΔoriC oriZ^+^* cells as long as the replication fork trap is active, demonstrating that the lethality of *ΔoriC oriZ^+^ ΔrecG* cells is caused by serious problems in the termination area.

To investigate whether this problem is related to the over-replication of the terminus area seen in the absence of RecG we generated a *ΔoriC oriZ^+^ ΔrecG priA300* construct. A *priA300* point mutation eliminates the helicase activity of PriA but leaves the replication restart activity intact (13). We demonstrated previously that this abolishes over-replication in cells lacking RecG (13,14). The synthetic lethality assay revealed that RecG is no longer essential (Figure 1B, panel x). Thus, it seems that the loss of viability in the absence of RecG is a consequence of over-replication or of some intermediate normally processed by RecG that triggers this over-replication.

### Over-replication within an ectopic termination area

**Figure 2.**
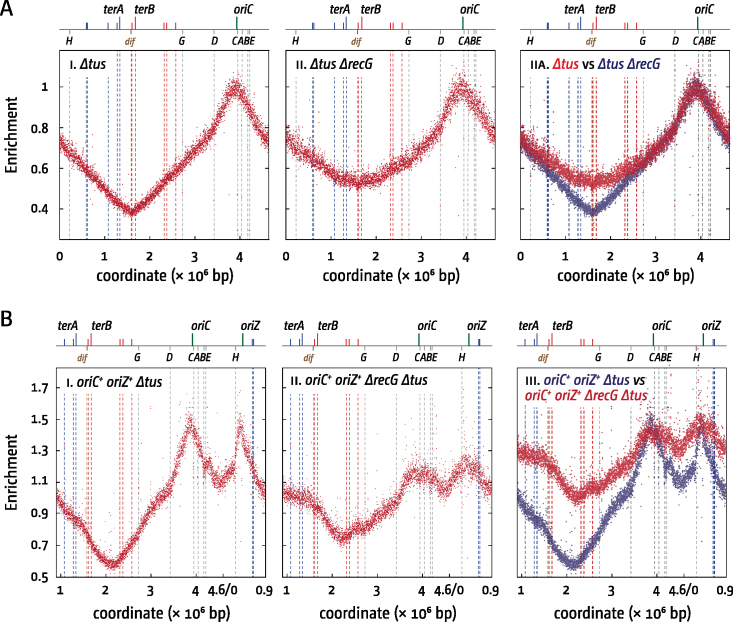
Over-replication in *E. coli ΔrecG* cells with one and two replication origins in the absence of functional *ter*/Tus replication fork traps. (**A**) Chromosomal marker frequency analysis of *E. coli Δtus* cells in the presence and absence of RecG helicase. The numbers of reads (normalised against reads for a stationary phase wild type control) are plotted against the chromosomal location. A schematic representation of the *E. coli* chromosome showing positions of *oriC* and *oriZ* (green line) and *ter* sites (above) as well as *dif* and *rrn* operons A–E, G and H (below) is shown above the plotted data. The strains used were N8227 (*Δtus*) and N7957 (*Δtus ΔrecG*). Data were re-plotted from (13). (**B**) Chromosomal marker frequency analysis of *oriC^+^ oriZ^+^ Δtus* cells in the presence and absence of RecG helicase. The strains used were RCe567 (*oriC^+^ oriZ^+^ Δtus*) and JD1135 (*oriC^+^ oriZ^+^ Δtus ΔrecG*).

Azeroglu and colleagues proposed recently that the over-replication seen in *ΔrecG* cells is a consequence of PriA-mediated assembly of replication fork complexes at branched DNA-structures generated after forks have been trapped at *ter*/Tus complexes within the normal termination area (30). This idea would be consistent with our finding that deleting *tus* restores viability to *ΔoriC oriZ^+^ ΔrecG* cells were it not for the fact that over-replication is still observed in a strain lacking both Tus and RecG, as is evident from the initial studies reported by Rudolph and colleagues (13). A re-analysis of their data revealed that while there is no longer a distinct peak of over-replication in *ΔrecG Δtus* cells, marker frequency is elevated over a broad region flanking the terminus area (Figure 2A). This is exactly what would be expected if the over-replication triggered in the absence of RecG is the result of some pathological event that is independent of *ter*/Tus traps and which establishes forks that then move back through the termination area. In the absence of Tus, these forks would no longer be restricted to the terminus region. Re-replication would spread out in the direction of *oriC*. Indeed the ability of *ΔrecG* cells to grow in the absence of origin firing depends on the absence of Tus, not its presence (13,14).

We examined the replication profile of *oriC^+^ oriZ^+^* cells lacking Tus or both Tus and RecG to see if there is a similar broadening effect of over-replication at an ectopic termination region. With RecG present both termination regions were clearly defined. In the absence of RecG, the replication profile was notably flattened, consistent with origin-independent replication occurring in both termination regions (Figure 2B). If the replication profiles of both backgrounds are aligned according to the *oriC* peak height, the over-replication in both termination areas becomes particularly obvious (Figure 2B, panel iii). To ensure that alignment via *oriC* peak heights is indeed justified we confirmed that doubling times for *oriC^+^ oriZ^+^ Δtus* in the presence and absence of RecG are very similar (26.6 ± 0.13 and 25.6 ± 0.75 min for *oriC^+^ oriZ^+^ Δtus* and *oriC^+^ oriZ^+^ Δtus ΔrecG*, respectively), confirming that the frequency of initiation is essentially unaffected by the absence of RecG.

**Figure 3.**
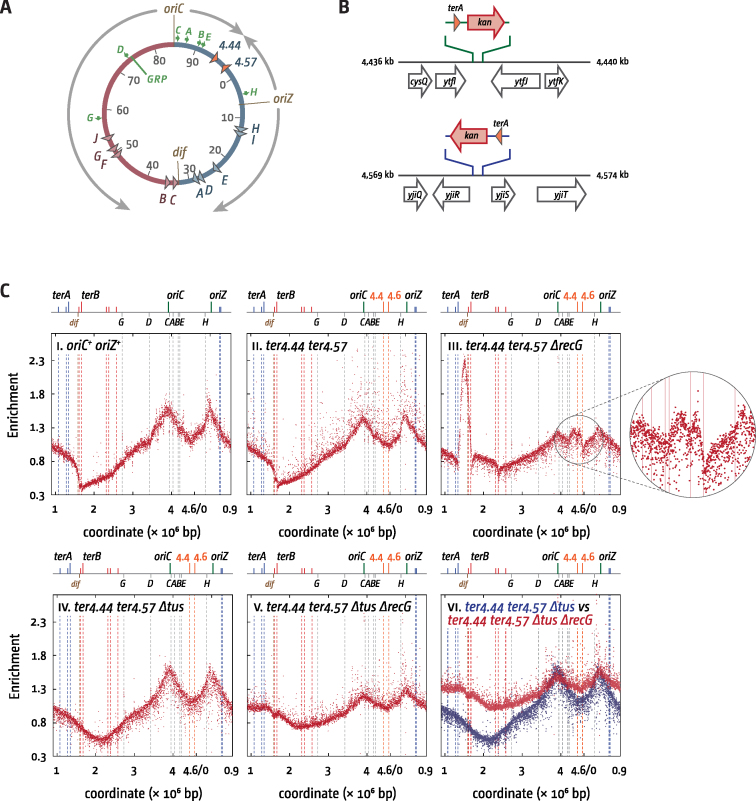
Replication profiles of *E. coli oriC^+^ oriZ^+^* cells with an ectopic replication fork trap in the presence and absence of RecG helicase. (**A**) Schematic representation of the chromosome of *oriC^+^ oriZ^+^* cells with additional *ter* sites integrated either side of the ectopic fork fusion area. (**B**) Schematic representation of the precise integration points of *terA* sites at the 4.44 and 4.57 Mb locations. (**C**) Chromosomal marker frequency analysis of *E. coli oriC^+^ oriZ^+^* and *oriC^+^ oriZ^+^ Δtus* cells with an ectopic replication fork trap in the presence and absence of RecG helicase. The numbers of reads (normalised against reads for a stationary phase wild type control) are plotted against the chromosomal location. A schematic representation of the *E. coli* chromosome showing positions of *oriC, oriZ* and *ter* sites (above) as well as *dif* and *rrn* operons A–E, G and H (below) is shown above the plotted data. The two ectopic *terA* sites are represented by orange lines. The strains used were RCe504 (*oriC^+^ oriZ^+^*), SLM1197 (*oriC^+^ oriZ^+^ ter4.44 ter4.57*), RCe714 (*oriC^+^ oriZ^+^ ter4.44 ter4.57 ΔrecG*), RCe745 (*oriC^+^ oriZ^+^ ter4.44 ter4.57 Δtus*) and RCe760 (*oriC^+^ oriZ^+^ ter4.44 ter4.57 Δtus ΔrecG*).

While the observed increase in marker frequency in the ectopic termination area of *oriC^+^ oriZ^+^ ΔrecG* cells is in line with the idea that the meeting and fusing of forks is the trigger for the over-replication observed (13), the absence of a clear peak makes the effect less visual than the over-replication observed in the native termination area. We therefore investigated whether a clear peak can be observed if a fork trap is reconstituted around the ectopic fork fusion point in double-origin cells (Figure 3A and B). The ectopic replication fork trap has very little effect on the replication profile of double-origin cells (Figure 3C, cf. panels i and ii). With ectopic *ter* sites present, the low point was close to *terA4.57* but there was no discontinuity in the replication profile similar to that seen in the normal termination area, making it unlikely that forks are blocked on a regular basis for extended periods. In the absence of RecG, this low point was replaced with a clear peak of over-replication (Figure 3C, panel iii). These data strongly suggest that the over-replication seen within the normal termination area in a *recG* single mutant is indeed a pathological consequence triggered by the meeting and fusion of converging replication fork complexes.

Over-replication in the ectopic termination area is not fully contained by the inserted *ter* sites, but bleeds towards *oriC* before it is impeded on encountering RNAP complexes head-on at *rrn* operons (Figure 3C, panel iii). It is tempting to speculate that this asymmetry is the same as in the native termination area, where the over-replication is blocked by *terB*, rather than *terC*, with little indication of any blocked forks at *terC* (13–14,30) (Supplementary Figure S1). There is no doubt that both *terC* and *terB* are fully proficient in blocking forks in the strains used in this study (4,35), suggesting that something might happen at or near *terC/terA4.44* that allows synthesis to proceed until forks are blocked at the next obstacle (*terB* or *rrn* operons).

Removing Tus eliminated the peaks observed within both the native and ectopic termination areas (Figure 3C, panel v) and an overlay of the *Δtus recG^+^* and *Δtus ΔrecG* profiles revealed the same extensive over-replication that spreads out in both directions as observed before (Figure 3C panel vi; Figure 2B, panel iii).

### The role of recombination in promoting initiation of pathological replication at sites of replisome fusion

**Figure 4.**
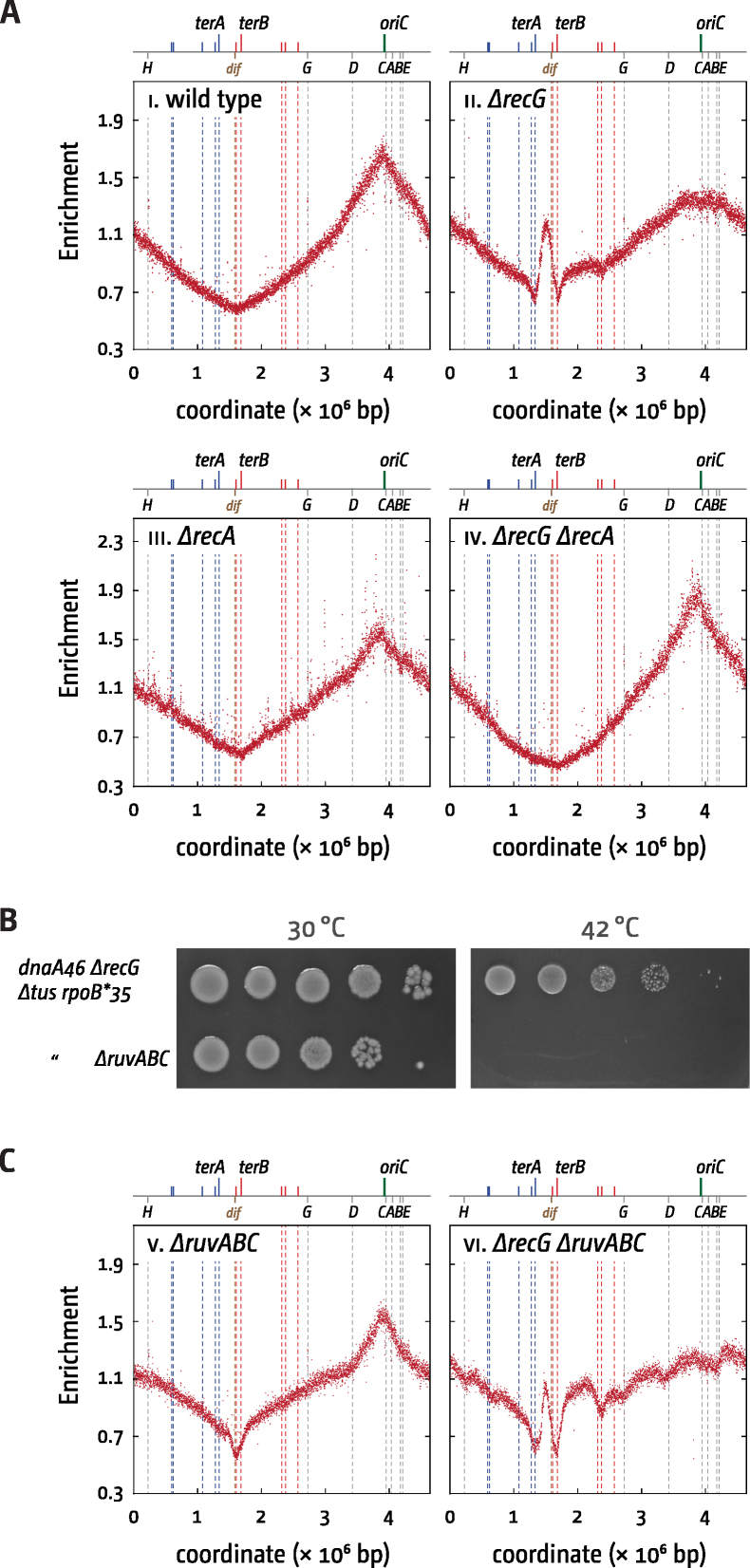
Over-replication in the termination area of *E. coli* cells in the presence and absence of recombination proteins. (**A**) Over-replication in the termination area of *ΔrecG* cells in the presence and absence of RecA recombinase. The number of reads (normalised against reads for a stationary phase wild type control) is plotted against the chromosomal location. A schematic representation of the *E. coli* chromosome showing positions of *oriC* and *ter* sites (above) as well as *dif* and *rrn* operons A–E, G and H (below) is shown above the plotted data. The strains used were MG1655 (wild type), N4560 (*ΔrecG*), AM1666 (*ΔrecA*) and RCe783 (*ΔrecA ΔrecG*). (**B**) Spot dilution assays to evaluate the ability of origin-independent growth of dnaA(ts) Δtus rpo* ΔrecG cells in the presence or absence of the Holliday junction resolvase RuvABC. The strains used were RCe268 (dnaA46 ΔrecG Δtus rpo*), and RCe526 (dnaA46 ΔrecG Δtus rpo* ΔruvABC). (**C**) Over-replication in the termination area of *ΔrecG* cells in the presence and absence of RuvABC. The strains used were JD1004 (*ΔruvABC*) and N4971 (*ΔrecG ΔruvABC*).

The data demonstrate that the over-replication of the terminus region seen in *ΔrecG* cells does not require forks to be blocked at *ter* sites. This is consistent with our previous studies demonstrating that *dnaA(ts) ΔrecG* cells can grow at restrictive temperature, but only if fork traps are eliminated by deletion of *tus* and conflicts between replication and transcription reduced by an rpo* mutation (13,14). Indeed, the quadruple mutant can tolerate deletion of the entire *oriC* area. We concluded that replication is maintained at restrictive temperature by the repeated establishment of new forks at structures generated every time forks fuse. Thus, growth of dnaA(ts) ΔrecG Δtus rpo* cells at 42°C can therefore serve as a proxy for this fork fusion-dependent, but origin-independent chromosome replication. We reported previously that eliminating the RecA or RecBCD recombinases eliminates this growth, supporting the notion that replication in this case depends on recombination. We also showed that the peak of over-replication in the native termination area in *ΔrecG* cells is absent in *ΔrecG ΔrecB* cells (13). The same proved true of *ΔrecG ΔrecA* cells (Figure 4A; see Supplementary Methods for the lack of a sharp *oriC* peak for the *ΔrecG* profile in panel ii).

In contrast, while eliminating the RuvABC Holliday junction resolvase from dnaA(ts) ΔrecG Δtus rpo* cells abolishes growth at restrictive temperature (Figure 4B), the replication profile of *ΔrecG ΔruvABC* cells revealed a clear peak of over-replication (Figure 4C). This suggests that dnaA(ts) tus rpo* ΔrecG cells lacking RuvABC fail to grow and divide at 42°C because they accumulate unresolved Holliday junctions rather than because of any failure in replication. Unresolved Holliday junctions would interfere with the segregation of the replicated chromosomes to daughter cells and indeed several studies have shown that mutations which trigger hyper-recombination such as *dam, polA* and *uvrD* are synthetically lethal with *ruv* (43–46). Taken together, these observations support the notion that origin-independent replication occurs via a recombinational mechanism that generates Holliday junctions. The requirement for RecBCD indicates that this recombination is initiated at duplex DNA ends.

### Replisome dynamics in cells that exhibit over-replication

**Figure 5.**
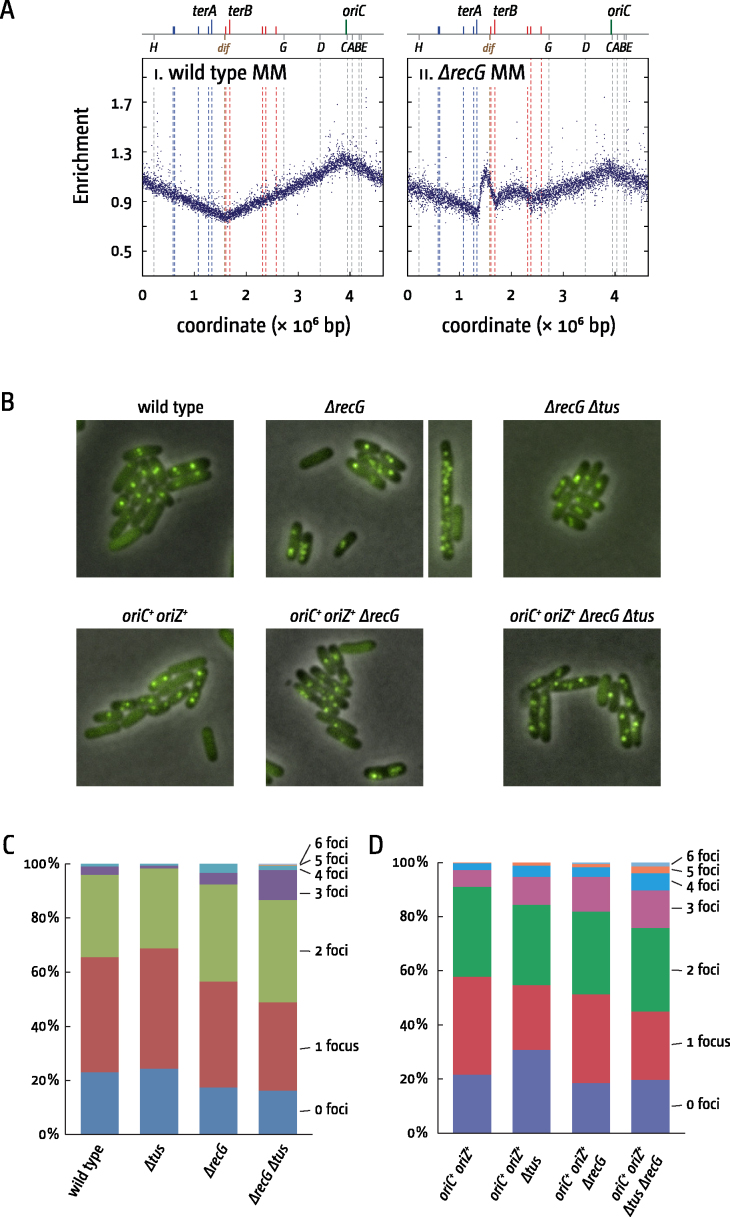
Replication dynamics in *ΔrecG* and *ΔrecG Δtus* cells in strain backgrounds with one or two replication origins. (**A**) Over-replication in the termination area of *ΔrecG* cells growing in M9 minimal salts with glucose. The numbers of reads (normalised against reads for a stationary phase wild type control) are plotted against the chromosomal location. A schematic representation of the *E. coli* chromosome showing positions of *oriC* and *ter* sites (above) as well as *dif* and *rrn* operons A–E, G and H (below) is shown above the plotted data. The strains used were MG1655 (wild type) and N4560 (*ΔrecG*). (**B**) Visualisation of replisomes (Ypet-DnaN) in wild type and *oriC^+^ oriZ^+^* backgrounds in the presence and absence of RecG helicase and a functional replication fork trap (*Δtus*). The larger image shows cells representative of the majority of *ΔrecG* cells observed. However, cells lacking RecG characteristically show a small fraction of filamented cells with aberrantly increased DnaN-foci numbers, as shown in the smaller image (see also Supplementary Figure S4). (**C**) Replisome numbers (YPet-DnaN) in the presence and absence of RecG helicase and a functional replication fork trap (*Δtus*). A total of 360 cells from 3 independent experiments were analysed per strain. Shown are the average focus counts per strain and focus class. For *ΔrecG* and *ΔrecG Δtus* counts all filamentous cells with aberrantly increased foci numbers were excluded from the analysis. The strains used were AS1062 (*ypet-dnaN*), RCe766 (*ypet-dnaN ΔrecG*), RCe777 (*ypet-dnaN Δtus*) and RCe768 (*ypet-dnaN Δtus ΔrecG*). See Supplementary Figure S3A for individual experiments. (**D**) Replisome numbers (YPet-DnaN) in *oriC^+^ oriZ^+^* cells in the presence and absence of RecG helicase and a functional replication fork trap (*Δtus*). The strains used were RCe749 (*oriC^+^ oriZ^+^ ypet-dnaN*), RCe773 (*oriC^+^ oriZ^+^ ypet-dnaN ΔrecG*), RCe758 (*oriC^+^ oriZ^+^ ypet-dnaN Δtus*) and RCe775 (*oriC^+^ oriZ^+^ ypet-dnaN Δtus ΔrecG*). See Supplementary Figure S3B for individual experiments.

Although slow growing, the high viability of dnaA(ts) ΔrecG Δtus rpo* cells at 42°C demonstrates that the molecular mechanism triggering origin-independent initiation of replication operates in the majority of the cell population, if not in every cell. However, being dependent on fork fusions, it cannot support the rapid growth that is made possible by re-firing of *oriC* before the previous cycle of replication is completed. To see if replication triggered by fork fusions in the terminus area of *ΔrecG* single mutants is equally widespread, we investigated whether these cells have increased numbers of replisomes. To do so, we used a strain in which the bright YFP derivative YPet was fused to the N-Terminus of the β-sliding clamp, encoded by the *dnaN* gene (47). We exploited the β-sliding clamp because it remains bound to DNA for some time after the replisome has passed (48,49) and might increase the likelihood of detecting short-lived bouts of over-replication in the terminus area. Cells were grown in M9 minimal medium with 0.2% glucose (see Material and Methods for further details). Under these conditions, the replication profile shows a reduced *ori/ter* ratio relative to that observed in broth-grown cells, but over-replication is still clearly evident in the absence of RecG (Figure 5A). We counted the number of fluorescent foci per cell (a minimum of 350 cells were counted from three independent experiments) (Figure 5B and C). We predicted that any additional replication in the terminus area would be reflected in increased numbers of fluorescent foci per cell.

In wild type cells we observed ∼20% of cells without any foci, indicative of one active round of synthesis having been completed while the next round has not yet started. ∼42% of cells showed a single focus and just over 30% showed two foci. Cells with more than two fluorescent foci were rare (<4%) (Figure 5C), highlighting that under these conditions rounds of chromosome duplication are essentially not overlapping. In *ΔrecG* cells we did observe a mild increase in the number of cells with two, three and four foci, while cells with zero and one focus were decreased. However, these changes were very mild (Figure 5B and C). In addition we observed a class of filamented cells with drastically increased foci numbers (Figure 5B). The uncontrolled amplification of limited chromosomal areas was observed before in *ΔrecG* cells suffering from genotoxic insult (50), suggesting that this particular class of cells might be suffering from the consequences of spontaneous DNA damage.

**Figure 6.**
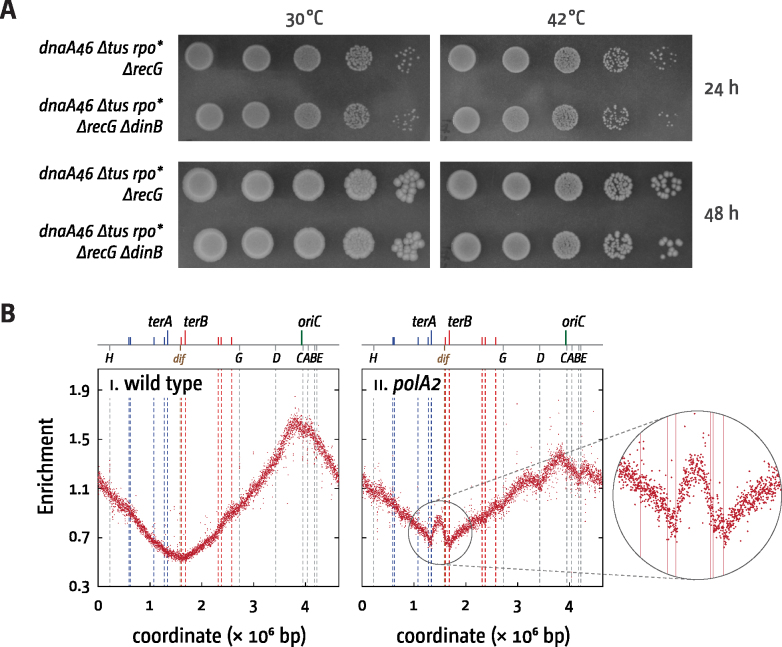
Impact of DNA polymerases IV and I on over-replication in the termination area. (**A**) Spot dilution assays to evaluate the ability of origin-independent growth of dnaA(ts) Δtus rpo* ΔrecG cells in the presence or absence of DNA polymerase IV. The strains used were RCe268 (dnaA46 ΔrecG Δtus rpo*), and JD1435 (dnaA46 ΔrecG Δtus rpo* ΔdinB). (**B**) Chromosomal marker frequency analysis of *E. coli* cells in the presence and absence of functional DNA polymerase I. The numbers of reads (normalised against reads for a stationary phase wild type control) are plotted against the chromosomal location. A schematic representation of the *E. coli* chromosome showing positions of *oriC* and *ter* sites (above) as well as *dif* and *rrn* operons A–E, G and H (below) is shown above the plotted data. The strains used were MG1655 (wild type) and JD1132 (*polA2*).

One explanation for the lack of increase in replisome numbers in *ΔrecG* cells could be that over-replication in the termination area is caused by DNA synthesis without the use of a β-sliding clamp. As the β-sliding clamp is the main processivity factor for DNA replication (51) this type of synthesis would not be processive enough for long stretches of chromosomal replication, but it might contribute at least in part to the over-replication observed. The two most obvious candidates would be DNA polymerase I (encoded by *polA*) and DNA polymerase IV (encoded by *dinB*), as genetic interactions of these two polymerases with *recG* were reported before. The combination of *ΔrecG* and a *polA2* allele, which encodes a protein retaining only the 5′ to 3′ exonuclease activity of pol I (46), was shown to be synthetically lethal (52), and levels of pol IV were significantly increased in *ΔrecG* cells (53). To test whether pol IV might contribute to the over-replication in *ΔrecG* cells we determined whether dnaA(ts) ΔrecG Δtus rpo* cells are able to grow at restrictive temperature in the absence of pol IV. As shown in Figure 6A, growth at restrictive temperature is barely affected by *ΔdinB*. The average of the difference of growth at 42°C/growth at 30°C for dnaA(ts) ΔrecG Δtus rpo* and dnaA(ts) ΔrecG Δtus rpo* ΔdinB from 4 experiments is just over 10%. Thus, it appears that DNA polymerase IV has little to do with the over-replication observed in the absence of RecG, in line with the reported rather specific role of pol IV in the postreplicative translesion synthesis in gaps behind the replisome (54). The role of pol I cannot be assessed in the same way because of the lethality of the *polA2* allele and *ΔrecG* (52). However, it was suggested before that *polA* cells show over-replication in the termination area (18), a result that our replication profiles confirmed (Figure 6B). Given that the absence of fully functional polymerase I itself causes over-replication of the termination area in itself it seems unlikely that the over-replication in *ΔrecG* cells is dependent on DNA polymerase I. The synthetic lethality in particular suggests the opposite, similar to the reported synthetic lethality of *ΔrecG* with other mutations that trigger origin-independent replication (55,22). There is little reason to believe that DNA polymerases II and V might be involved in the over-replication in *ΔrecG* cells, and indeed introduction of *polB* or *umuDC* alleles into dnaA(ts) ΔrecG Δtus rpo* cells did not change their ability to grow at 42°C (Supplementary Figure S2).

So why do *ΔrecG* cells show such a minor increase in the number of DnaN foci when there is a pronounced peak of over-replication in the termination area, especially in double origin constructs? It would fit with a model in which over-replication of the termination area is restricted to a minority of the cells, those where forks meet and fuse at a *ter* site, but as we have argued, this is not compatible with the robust viability of dnaA46 ΔrecG Δtus rpo* cells at 42°C and it also does not fit with the excessive level of over-replication observed in *oriC^+^ oriZ^+^ ΔrecG* cells (Figure 3C, iii).

Another explanation would be the disassembly of the replisomes responsible for the over-replication as they run into *ter*/Tus complexes flanking the termination area. It was recently reported that replisomes remain stably bound at *ter*/Tus complexes (56). However, both *in vitro* and *in vivo* measurements of fork stability at obstacles such as supercoiling and repressor-operator complexes suggest a limited half-life of 4–6 min (57–59). There is no indication that *ter*/Tus complexes would stabilize replisomes, supporting the idea that replication fork complexes might disassemble after a limited period of time. If true then the deletion of *tus* should lead to an increase in the number of fluorescently tagged replisomes in *ΔrecG* cells, as forks initiated in the termination area are able to proceed. Indeed, in *ΔrecG Δtus* cells we found a shift towards cells with higher numbers of foci, while foci numbers in a *Δtus* single mutant were, if anything, reduced in comparison to wild type cells (Figure 5C; see Supplementary Figure S3 for individual experiments).

Since levels of over-replication are much exacerbated in the absence of RecG in cells with an additional ectopic replication origin (13), foci numbers should be increased further in *oriC^+^ oriZ^+^ ΔrecG* cells. Indeed, *oriC^+^ oriZ^+^ ΔrecG* cells and particularly *oriC^+^ oriZ^+^ Δtus ΔrecG* cells showed elevated foci numbers (Figure 5D; see Supplementary Figure S3 for individual experiments), in line with the idea that replisomes arrested at *ter*/Tus complexes might be disassembled after a certain period of time.

Could the absence of any obvious increase in the number of DnaN foci in *ΔrecG* cells be due to the limited resolution of the conventional fluorescence microscopy we used? Given the relatively small size of the termination area, forks arrested at *terC* and *terA* might appear as a single focus. As deletion of *tus* would allow these forks to proceed and move further apart, they might then form separate foci, explaining the increase of focus numbers particularly in *ΔrecG Δtus* cells. While we cannot entirely exclude this possibility, we think it cannot be solely responsible for the similarity of foci numbers in wild type and *ΔrecG* cells. In wild type cells rounds of DNA synthesis are non-overlapping in M9 minimal medium with 0.2% glucose. Replisomes are disassembled upon completion of DNA replication and new forks are initiated at the segregated copies of *oriC*, in line with our data showing that wild type cells show either zero (completed synthesis), one (ongoing synthesis) or two foci (initiation of synthesis at segregated origins). The persistence of foci in the termination area should lead to cells with three foci, as new forks would still be assembled at *oriC*. However, three foci are observed rarely, both in wild type and *ΔrecG* cells (Figure 5C). Additional support for the idea that replisomes might be disassembled comes from the observation that in double origin cells the deletion of *tus* results in an increase of cells with zero, three, four and five foci, while the number of cells with one and two foci is reduced (Figure 5D). In contrast, a *Δtus* single mutant shows a mild reduction of foci numbers (Figure 5C). The biggest difference between *Δtus* and *oriC^+^ oriZ^+^ Δtus* cells is that replication forks coming from *oriZ* travelling clockwise will reach the replication fork trap much earlier than forks coming from *oriC* travelling counter clockwise. Thus, a high proportion of forks will be arrested at *ter*/Tus complexes (4,35). The fact that the deletion of *tus* leads to an increase in the number of fluorescent foci in double-origin cells is in line with the idea that replisomes blocked at *ter*/Tus complexes disassemble after a relatively limited period, as observed in previous studies (57–59).

The most obvious conclusion that can be drawn from these data is perhaps that the increase in the number of replisomes per cell does not appear to directly reflect the level of origin-independent replication seen in the absence of RecG. If over-replication initiates in the cell population at large, as is clearly indicated by the high viability of dnaA46 ΔrecG Δtus rpo* cells at 42°C, then our data are in line with the idea that a replisome held up at a *ter*/Tus complex tends to dissociate after a limited period, a likelihood that increases the longer the fork is held up. If true, then replication will terminate when the converging fork complex meets a ‘naked’ fork rather than when it meets and fuses with another fork complex. If the naked fork is exposed to DNA processing enzymes before the arrival of the converging fork, the resulting fusion may well have pathological consequences in the absence of RecG that are reflected in the observed dramatic sequence amplification.

## DISCUSSION

In this study we have further defined the molecular role that RecG plays in preventing over-replication in the termination area of the chromosome and the molecular mechanisms involved in triggering this over-replication. The ability of RecG protein to unwind R-loops (60,61) has previously led to the suggestion that in the absence of RecG R-loops persist in defined chromosomal locations such as the termination area, which trigger origin-independent DNA synthesis (24,27–28,55). We have demonstrated previously that the molecular mechanism for initiation of synthesis at R-loops in cells lacking RNase HI differs from the mechanisms responsible for over-replication in the termination area in *ΔrecG* cells (14), making it unlikely that the ability of RecG to dissociate R-loops *in vivo* is key to prevent the over-replication in the termination area (14). We suggest instead that the over-replication is likely to stem from pathological events initiated in areas where replication forks fuse (13). The data presented in this study strongly support this notion. We found that the over-replication detectable in an ectopic termination area created by the presence of a second copy of the origin can be converted to a sharply defined peak (Figure 3). This observation essentially rules out the idea that DNA synthesis is initiated via an origin-like activity, such as a defined hotspot for R-loop formation (24) or a cryptic replication origin normally suppressed by RecG.

We exploited fluorescent microscopy to see if the over-replication of DNA seen in the absence of RecG could be detected at the level of single cells. As already discussed, these studies met with limited success. Differences in the detected number of foci of β-sliding clamp between the wild type and various mutant strains analyzed were generally rather minor, with the only substantial trend being the increase in the number of cells with 3–6 foci when RecG was eliminated. This trend was increased by the additional elimination of Tus, and further exacerbated in a strain with two active copies of the origin (Figure 5C) where over-replication in the absence of RecG is dramatically increased (13) (Figure 3).

The presence of a subpopulation with dramatically increased foci numbers raised the possibility that the over-replication detected in the absence of RecG might be a feature of only a small sub-set of cells in the population sampled. Such a minority of cells was recently shown to be responsible for the sharp loss of sequences corresponding to the terminus area in the replication profile of a *recB* mutant strain (62). However, we observed no correlation between the level of over-replication in the termination area and the number of cells with aberrantly increased replisome foci (cf. Figure 4A ii, 3C iii and Supplementary Figure S4). In addition, both the 60% value we previously reported (13) for the viability of dnaA(ts) ΔrecG Δtus rpo* cells at the restrictive temperature as well as the extreme levels of over-replication in the termination area in *oriC^+^ oriZ^+^ ΔrecG* cells tends to rule out that a similar minority of cells accounts for the replication profile of a population of *ΔrecG* cells. Perhaps the strongest argument against a minority of cells is the lethality of *ΔoriC oriZ^+^ ΔrecG* cells (Figure 1). The fact that the lethality is suppressed by the deletion of *tus* demonstrates that it must be caused by some pathological event in the termination area, and the suppression by *priA300* supports the idea that it is the over-replication that is responsible. If the over-replication that leads to lethality in *ΔoriC oriZ^+^ ΔrecG* cells was restricted to a small fraction of cells we would expect the rest to be able to form white colonies, even though they might be smaller in size. The dramatic reduction of white colonies strongly suggests that the pathology must occur in the majority of cells.

Could over-replication be triggered at forks stalled at *ter*/Tus complexes, as suggested (30)? This would provide an explanation as to why over-replication is strongly increased in *oriC^+^ oriZ^+^ ΔrecG* cells, as *oriZ* forks are initiated much closer to the replication fork trap, dictating that a substantial fraction of forks will be stalled (Figure 1). However, the ability of *ΔrecG* cells to grow in the absence of origin firing depends on the ***absence*** of Tus, not its presence (13,14). In addition, in this study we show that the number of DnaN foci indicative of active replication forks is increased in *ΔrecG* and *oriC^+^ oriZ^+^ ΔrecG* cells in particular if *tus* is deleted (Figure 5). Together with the flattening of replication profiles observed in *ΔrecG Δtus* and *oriC^+^ oriZ^+^ ΔrecG Δtus* cells (Figures 2 and 3) this rules out that Tus is essential for the over-replication observed. This fits with the observation that both over-replication in the termination area and aberrant replication intermediates in R1 plasmid replication were found upon deletion of *tus* (15,18).

But why is over-replication so strongly exacerbated once an ectopic replication origin is present in the chromosome (Figure 3) (13)? Given that Tus protein is not a key factor there must be a different explanation. Another significant difference between wild type and *oriC^+^ oriZ^+^* cells is how long forks are stalled at *ter*/Tus complexes. Moolman *et al*. have shown that the replisome reaching *terC* in *oriC^+^ oriZ^+^* cells remains stably bound (56). However, our own data are consistent with the idea that at least a fraction of replisomes might be disassembled (Figure 5), in line with measurements of replisome stability *in vitro* and *in vivo* at different obstacles such as supercoiling and repressor-operator complexes, which suggest a half-life of 4–6 min for the stable arrest of replication forks (57–59). Thus, replisomes might well be stably arrested for a limited period before they dissociate, and it might be precisely the balance between arrested and dissociated replisomes that is an important factor. In cells lacking RecG, forks are also likely to be arrested at *ter*/Tus complexes for extended periods of time. Over-replication triggered in the termination area in the absence of RecG will proceed until it is blocked by *ter*/Tus complexes. If this over-replication is triggered by fork fusion events the stalled forks will have to wait for considerable periods until another round of DNA synthesis coming from *oriC* reaches the arrested forks. Indeed, Azeroglu *et al*. reported the accumulation of RecA in the termination area of *ΔrecG* cells, with highest levels accumulating at *chi* sites in active orientation closest to where forks are blocked (23,30). This provides a strong indication that the blocked forks are processed by RecBCD and RecA. However, it is likely that access by RecBCD will only be possible once the replisome is moved out of the way.

**Figure 7.**
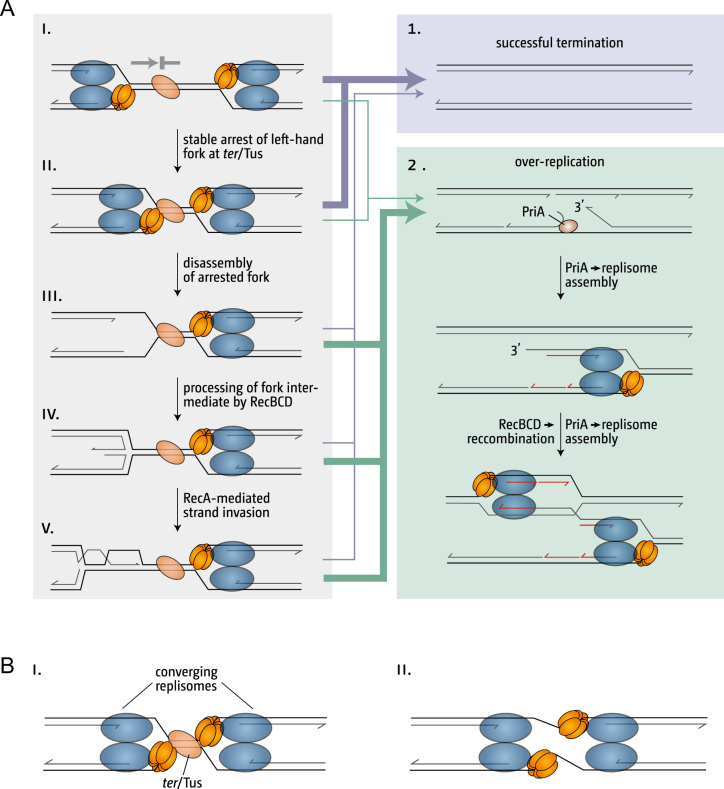
Over-replication in cells lacking RecG helicase can be modulated by the stable arrest of replisomes at *ter*/Tus complexes. (**A**) Replication fork fusion outcomes after one fork is arrested at a *ter*/Tus complex. The *ter*/Tus complex shown is in blocking orientation for forks coming from the left. Thickness of the arrows shown corresponds with the proposed likelihood of the chosen pathway. Blue arrows represent the situation in wild type cells, while green arrows represent the situation in cells with defects in processing fork fusion intermediates, such as *ΔrecG*. See text for further details. (**B**) Final stages of replication fork fusion reactions in the presence and absence of a *ter*/Tus complex. See text for further details.

In wild type cells the stable arrest of replisomes at *ter*/Tus complexes might be a rather important feature, as this will actively prevent the processing of the stalled forks while the second fork is likely to reach the termination area soon after (Figure 7i & ii). We have suggested that the displacement of a 3′ flap is a particular risk following collision of two replisomes (12,15,18). A collision between the leading strand polymerase and the helicase of the opposing fork might well cause the partial unwinding of the nascent leading strand in the active centre of the polymerase. This risk might be considerably reduced if one entire replisome is paused via the arrest of the helicase at a *ter*/Tus complex, as both Tus and the arrested helicase will act as a ‘buffer’ that stops progression of the opposing helicase (Figure 7) (3). In contrast, in *oriC^+^ oriZ^+^* cells replisomes remain stalled for much longer, increasing the likelihood of fork disassembly (Figure 7iii) and subsequent processing (Figure 7iv & v). In line with this idea we observed that while *terC* and *terB* appear to form pause sites with equal strength in *oriC^+^ oriZ^+^* cells, forks proceed beyond *terC* more frequently in *ΔoriC oriZ^+^* cells in which the fork coming from *oriZ* and traversing counter-clockwise has to replicate 75% of the chromosome (4,35), in line with the initial characterization of *ter* as being strong pause sites, rather than absolute blocks (3,63–64). Similarly, we found that if *ΔrecG* cells are grown in minimal medium, the over-replication in the termination area appears to overcome *terB* to some extent (Supplementary Figure S5). This observation is in line with the idea that because of the reduced replication initiation frequency of cells grown in minimal salts, forks initiated within the termination area will be blocked at *ter*/Tus complexes for longer until they are reached by a fork coming from *oriC* (Supplementary Figure S5). Thus, our observations are in line with the idea that a longer delay results in a higher chance of processing, which eventually will enable a fork to proceed through the *ter*/Tus barrier, as observed (56). Thus, we suggest that replisomes remain initially stably associated at *ter*/Tus and that fork fusion events between a moving and a stably arrested replisome is less likely to trigger over-replication in the absence of RecG (Figure 7ii & A). In contrast, a fusion event between a moving replisome and a dissociated ‘naked’ fork or a fork processed by other protein factors might result in origin-independent replication (Figure 7iii–v & B). The exacerbation of over-replication in *oriC^+^ oriZ^+^ ΔrecG* and in *ΔoriC oriZ^+^ ΔrecG* cells would therefore be caused by the much elongated periods for which replisomes are stalled at *ter*/Tus complexes, causing essentially every fork fusion event to trigger re-replication in the vicinity of the fork fusion. A time-dependent dissociation of replisomes stalled *ter*/Tus complexes could also explain the discrepancy between our own data and the data by Moolman and colleagues (Figure 5) (56).

But how do highly elevated levels of over-replication cause lethality in *ΔoriC oriZ^+^ ΔrecG* cells? *oriC^+^ oriZ^+^ ΔrecG* cells appear to have little trouble coping with the increased levels of over-replication in the termination area (Figure 1). We suggest that this is directly linked to the number of recombination events. Our observation that dnaA(ts) Δtus rpo* ΔrecG ΔruvABC cells cannot grow at restrictive temperature even though over-replication is still observed in the termination area (Figure 4) suggests that unresolved recombination intermediates accumulate and prevent successful chromosome segregation in *ΔrecG* cells. In *ΔoriC oriZ^+^ ΔrecG* cells the number of recombination events within a relatively limited area of the chromosome might become so high that the cells are unable to survive. Deletion of *tus* would allow forks to proceed, thereby leading to a wider area where recombination is occurring. In addition, with less forks actively stalled at *ter*/Tus complexes, the deletion of *tus* would reduce levels of over-replication, as it would mainly occur at fork fusion locations and likely at similar levels as observed in *ΔrecG* cells (Figures 4 and 7).

The notion that the stable arrest of replisomes at *ter*/Tus complexes is an important aspect of the functionality of a replication fork trap would indeed add to our understanding of the physiological role of the termination area. We and others have suggested before that the main function of the replication fork trap might be to safely contain over-replication triggered if fork fusion events are not adequately processed (3–4,13,18,32). The stable arrest of replisomes at *ter*/Tus complexes might be yet another mechanism that prevents over-replication in the termination area, together with an increasing number of proteins such as RecG, PriA, RecBCD, Exo I, Exo VII, SbcCD and Pol I (4,13–15,18,25,29,22). Thus, *ter*/Tus complexes might not only prevent fork movement but specifically maintain replication fork integrity until DNA replication can terminate. Given that forks arrested at *ter*/Tus complexes are eventually disassembled it will not be beneficial to arrest forks on a regular basis at *ter*/Tus complexes and indeed it appears that forks terminate mostly away from *ter*/Tus (4,65). Forks will only reach *ter*/Tus complexes if the second fork gets delayed for a longer period of time. However, given that forks stalled at obstacles can efficiently be restarted (66,67) the stable arrest of the second replisome might well be the safest way of bringing DNA replication to a successful and accurate conclusion.

## DATA AVAILABILITY

All relevant raw sequencing data can be accessed at the European Nucleotide Archive (http://www.ebi.ac.uk/ena/data/view/PRJEB25595)

## Supplementary Material

Supplementary Data
