# Supplementary material for: Chromosomal over-replication in Escherichia coli recG cells is triggered by replication fork fusion and amplified if replichore symmetry is disturbed
Source: Nucleic Acids Res. 2018 Jun 30;46(15):7701–15. doi: 10.1093/nar/gky566 (PMC6125675; doi:10.1093/nar/gky566)
Supplement: Supplementary Data [file gky566_supplemental_files.pdf]

Supplementary Information

Chromosomal over-replication in *Escherichia coli recG* cells is triggered by replication fork fusion and amplified if replichore symmetry is disturbed

Sarah L. Midgley-Smith[1]†, Juachi U. Dimude[1]†, Toni Taylor[1], Nicole M. Forrester[1], Amy L. Upton[1], Robert G. Lloyd[2] and Christian J. Rudolph[1]*

†Both authors contributed equally to this work

*Corresponding author: christian.rudolph@brunel.ac.uk

1 Division of Biosciences, College of Health and Life Sciences, Brunel University London, Uxbridge, UB8 3PH, UK

2 Medical School, Queen's Medical Centre, Nottingham University, Nottingham NG7 2UH

SUPPLEMENTARY TABLES

Supplementary Table 1: *Escherichia coli* K-12 strains

Strain number	Relevant Genotype[a]	Source
**General P1 donors**		
WX297	AB1157 oriZ-<kan>	(1)
RRL190	AB1157 <kan>-ypet-dnaN	(1)
RW82	ΔumuCD595::cat	(2)
**MG1655 derivatives**		
MG1655	F^{-} rph-1	(3)
AM1417	ΔpyrE::dhfr	(4)
AM1655	ΔrecG::apra	(5)
AM1666	ΔrecA::apra	(6)
AM1775	Δtus::cat	(7)
AM1780	ΔpolA2::dhfr	(4)
AM1783	ΔlacIZYA<> ΔpolA2::dhfr	TB28 × P1.AM1780 to Tm^{r}
AM1968	ΔlacIZYA<> ΔpolA2::dhfr pAM475[b]	AM1783 × pAM475 to Ap^{r}
AS1062	<kan>-ypet-dnaN	MG1655 × P1.RRL190 to Km^{r}
AU1015	ΔlacIZYA<> ΔrecG::apra	Plasmid-free derivative of JJ1119
AU1216	$recG^{wt}$-kan	ALU and RGL, unpublished
AU1238	ΔlacIZYA<> ΔpolA2::dhfr $recG^{wt}$-kan pAM475	AM1968 × P1.AU1216 to Km^{r} Ap^{r}
JD1004	ΔruvABC::cat	MG1655 × P1.N4971 to Cm^{r}
JD1132	ΔpolA2::dhfr	MG1655 × P1.AU1238 to Tm^{r}
JD1135	oriZ-<cat> tus1::dhfr ΔrecG::apra	RCe567 × P1.AU1015 to $Apra^{r}$
JD1234	rpoB*35 ΔlacIZYA<> oriZ-<cat> ΔrecG::apra tus1::dhfr pJJ100[b]	RCe612 × P1.N6798 to Tm^{r}
JD1246	rpoB*35 ΔlacIZYA<> oriZ-<cat> ΔrecG::apra tus1::dhfr ΔoriC::kan[c] pJJ100	JD1234 × P1.RCe576 to Km^{r}
JD1248	rpoB*35 ΔlacIZYA<> oriZ-<cat> ΔrecG::apra ΔoriC::kan pJJ100	RCe612 × P1.RCe576 to Km^{r}
JD1154	ΔlacIZYA<> oriZ-<cat> tus1::dhfr	RCe544 × P1.N6798 to Tm^{r}
JD1284	ΔlacIZYA<> oriZ-<cat> tus1::dhfr pJJ100	JD1154 × pJJ100 to Ap^{r}
JD1280	ΔlacIZYA<> oriZ-<cat> pJJ100	RCe544 × pJJ100 to Ap^{r}
JD1285	ΔlacIZYA<> oriZ-<cat> ΔrecG::apra pJJ100	JD1280 × P1.AU1015 to $Apra^{r}$ Ap^{r}
JD1287	ΔlacIZYA<> oriZ-<cat> tus1::dhfr ΔrecG::apra pJJ100	JD1284 × P1.AU1015 to $Apra^{r}$ Ap^{r}
JD1292	ΔlacIZYA<> oriZ-<cat> tus1::dhfr ΔrecG::apra ΔoriC::kan pJJ100	JD1287 × P1.RCe576 to Km^{r} Ap^{r}
JD1293	ΔlacIZYA<> oriZ-<cat> ΔrecG::apra ΔoriC::kan pJJ100	JD1285 × P1.RCe576 to Km^{r} Ap^{r}
JJ1060	priA300 ΔlacIZYA<>	(4)
JJ1076	priA300 ΔlacIZYA<> pJJ100	JJ1060 × pJJ100 to Ap^{r}

JD1303	*priA300 ΔlacIZYA<>*	Plasmid-free derivative of JJ1076
JD1314	*priA300 ΔlacIZYA<>* pAM374	JD1303 × pAM374 to Ap^{r}
JD1319	*priA300 ΔlacIZYA<> oriZ-<cat>* pAM374	JD1314 × P1.RCe544 to Cm^{r}
JD1421	*priA300 ΔlacIZYA<> oriZ-<cat> ΔrecG::apra* pAM374	JD1319 × P1.AU1015 to Apra^{r} Ap^{r}
JD1430	*priA300 ΔlacIZYA<> oriZ-<cat> ΔrecG::apra*	Plasmid-free derivative of JD1421
JD1431	*priA300 ΔlacIZYA<> oriZ-<cat> ΔrecG::apra* pJJ100	JD1430 × pJJ100 to Ap^{r}
JD1432	*priA300 ΔlacIZYA<> oriZ-<cat> ΔrecG::apra ΔoriC::kan* pJJ100	JD1431 × P1.RCe576 to Km^{r}
JD1435	*rpoB*35 ΔrecG::apra Δtus::cat tnaA::*Tn*10 dnaA46 ΔdinB::zeo*	RCe268 × P1.N5254 to Zeo^{r}
JJ1119	*ΔlacIZYA<> ΔrecG::apra* pJJ100	(4)
JJ1359	*ΔlacIZYA dam1::kan ΔrecG::apra tus1::dhfr*	(7)
JD1440	*rpoB*35 ΔrecG::apra dnaA46 tnaA::*Tn*10 tus1::dhfr ΔpolB::spc/str*	RCe519 × P1.N6146 to Str^{r}
JD1441	*rpoB*35 ΔrecG::apra dnaA46 tnaA::*Tn*10 tus1::dhfr ΔumuCD595::cat*	RCe519 × P1.RW82 to Cm^{r}
N4560	*ΔrecG265::cat*	(8)
N4971	*ΔrecG263::kan ΔruvABC::cat*	(9)
N5254	*ΔdinB::zeo*	(6)
N5925	*rpoB*35 ΔlacIZYA<>*	(10)
N6146	*ΔpolB::spc/str*	(11)
N6798	*ΔrecG265::cat tus1::dhfr*	N4560 × P1.JJ1359 to Tm^{r}
N7957	*ΔrecG::apra Δtus::cat*	AM1655 × P1.AM1775 to Cm^{r}
N8227	*Δtus::cat*	MG1655 × P1.AM1775 to Cm^{r}
RCe267	*rpoB*35 Δtus::cat tnaA::*Tn*10 dnaA46*	(7)
RCe268	*rpoB*35 ΔrecG::apra Δtus::cat tnaA::*Tn*10 dnaA46*	(7)
RCe452	*ΔrecG::apra*	MG1655 × P1.AU1015 to Apra^{r}
RCe504	*oriZ-<cat>*	(12)
RCe519	*rpoB*35 ΔrecG::apra dnaA46 tnaA::*Tn*10 tus1::dhfr*	RCe268 × P1.N6798 to Tm^{r} Cm^{s}
RCe526	*rpoB*35 ΔrecG::apra dnaA46 tnaA::*Tn*10 tus1::dhfr ΔruvABC::cat*	RCe519 × P1.N4971 to Cm^{r}
RCe544	*ΔlacIZYA<> oriZ-<cat>*	TB28 × P1.WX269 to Cm^{r}
RCe567	*oriZ-<cat> tus1::dhfr*	(12)
RCe576	*rpoB*35 oriZ-<cat> tus1::dhfr ΔoriC::kan*	(12)
RCe585	*rpoB*35 ΔlacIZYA<> oriZ-<cat>*	N5925 × P1.RCe544 to Cm^{r}
RCe610	*rpoB*35 ΔlacIZYA<> oriZ-<cat> ΔrecG::apra*	RCe585 × P1.AU1015 to Apra^{r}
RCe612	*rpoB*35 ΔlacIZYA<> oriZ-<cat> ΔrecG::apra* pJJ100	RCe610 × pJJ100 to Ap^{r}
RCe714	*ter4.44<> oriZ-<cat> ter4.57-<kan> ΔrecG::apra*	SLM1197 × P1.AU1015 to Apra^{r}

RCe745	*ter4.44<> oriZ-<cat> ter4.57-<kan> tus1::dhfr*	SLM1197 × P1.N6798 to Tm^{r}
RCe749	*oriZ-<cat> <kan>-ypet-dnaN*	RCe504 × P1.AS1062 to Km^{r}
RCe758	*oriZ-<cat> tus1::dhfr <kan>-ypet-dnaN*	RCe567 × P1.AS1062 to Km^{r}
RCe760	*ter4.44<> oriZ-<cat> ter4.57-<kan> tus1::dhfr ΔrecG::apra*	RCe745 × P1.AU1015 to Apra^{r}
RCe766	*ΔrecG::apra <kan>-ypet-dnaN*	RCe452 × P1.AS1062 to Km^{r}
RCe768	*ΔrecG::apra Δtus::cat <kan>-ypet-dnaN*	N7957 × P1.AS1062 to Km^{r}
RCe773	*oriZ-<cat> <kan>-ypet-dnaN ΔrecG::apra*	RCe749 × P1.RCe766 to Apra^{r}
RCe775	*oriZ-<cat> tus1::dhfr <kan>-ypet-dnaN ΔrecG::apra*	RCe758 × P1.RCe766 to Apra^{r}
RCe777	*Δtus::cat <kan>-ypet-dnaN*	AM1775 × P1.AS1062 to Km^{r}
RCe783	*ΔrecG265::cat ΔrecA::apra*	N4560 × P1.AM1666 to Apra^{r}
SLM1193	*ter4.44<> oriZ-<cat>*	SLM1192 × P1.RCe544 to Cm^{r}
SLM1192	*ter4.44<>*	SLM1182 × pCP20 to Km^{s} (Ap^{s})
SLM1182	*ter4.44<kan>*	MG1655 × P1.SLM1113 to Km^{r}
SLM1115	*Δtus::cat ter4.57-<kan>*	This study (see Material & Methods)
SLM1113	*Δtus::cat ter4.44-<kan>*	This study (see Material & Methods)
SLM1197	*ter4.44-<> oriZ-<cat> ter4.57-<kan>*	SLM1193 × P1.SLM1115 to Km^{r}
TB28	*ΔlacIZYA<>*	(13)

a – Only the relevant additional genotype of the derivatives is shown. The abbreviations *amp, apra, kan, cat, dhfr* and *zeo* refer to insertions conferring resistance to ampicillin (Ap^{r}), apramycin (Apra^{r}), kanamycin (Km^{r}), chloramphenicol (Cm^{r}), trimethoprim (Tm^{r}) and zeocin (Zeo^{r}), respectively. '<>' indicates the use of *frt* sites, where *frt* stands for the 34 bp recognition site of the FLP/*frt* site-directed recombination system. Thus, <*kan*> refers to a kanamycin marker flanked by an *frt* site either side. <> refers a construct where the marker was lost due to the use of FLP recombinase, leaving a single *frt* scar (pCP20) (14).

b – pJJ100 is a derivative of pRC7 encoding *recG*$^{+}$ (6), while pAM475 is a pRC7 derivative encoding *polA*$^{+}$ (15). The wild-type *polA* coding sequence plus some 50 bp of upstream sequence was amplified by PCR from MG1655 using primers incorporating flanking *Apa*I restriction sites and then inserted into pRC7 at the *Apa*I site within *lacI*^{q}. pAM475 eliminates the sensitivity to UV light of a strain carrying a C-terminal deletion of polA (4), demonstrating that the cloned *polA*$^{+}$ gene is functional. pAM374 is a derivative of pRC7 encoding *priA*$^{+}$, cloned via *Eco*RI and *Hin*dIII behind the *lac* promoter, which allows expression control via IPTG (6).

c – *ΔoriC* refers to a replacement of the entire origin region (754 bp) including DnaA boxes and 13mers as well as the entire *mioC* gene by a kanamycin resistance cassette (7).

SUPPLEMENTARY METHODS

Marker frequency analysis by deep sequencing

Marker frequency analysis by Deep Sequencing was performed as described previously (12, 16, 17) with only minor modifications. Samples from cultures of a strain grown over night in LB broth were diluted 100-fold in fresh LB broth and incubated with vigorous aeration until an A_{600} reached 0.48 at 37°C to ensure they were in exponential growth conditions. Cultures were then diluted a second time 100-fold in pre-warmed fresh broth and grown again until an A_{600} of 0.48 was reached. Samples from these exponential phase cultures were flash-frozen in liquid nitrogen at this point for subsequent DNA extraction. For wild type incubation of the remaining culture was continued until several hours after the culture had saturated and showed no further increase in the A_{600}. A further sample (stationary phase) was frozen at this point. DNA was then extracted using the GenElute Bacterial Genomic DNA Kit (Sigma-Aldrich). Marker frequency analysis was performed using Illumina HiSeq 2500 sequencing (fast run) to measure sequence copy number. FastQC was used for a basic metric of quality control in the raw data. Bowtie2 was used to align the sequence reads to the reference. Samtools was used to calculate the enrichment of uniquely mapped sequence tags in 1 kb windows.

For presentation of the data as a marker frequency replication profile the raw read counts for each construct was divided by the average of all read counts across the entire genome to correct for the somewhat different absolute numbers of aligned reads in the various samples. The normalised read count values for each exponentially growing sample were then divided by the corresponding normalised read count value from a stationary (non-replicating) sample. This division "cleans" the raw data significantly, because data points which are outliers caused by technical aspects (precise sequence environment interfering with library preparation or similar issues) will be similarly distorted both in the exponential and the stationary samples. However, while true in principle, we have observed that there can be variations specifically in these noisy data points even within a single batch of samples processed in parallel. If the absolute sequence reads of the genome fragments causing the noisy data points in a sample are underrepresented in comparison to the same fragments in the stationary phase sample, then the division process described above causes all of these data points to skew below the position of the neighbouring data points. In contrast, if the absolute sequence reads of the fragments are higher than the sequence reads in the stationary control, then the same division process causes all of these data points to skew above the position of the neighbouring data points. An example of this effect can be seen in Figure 3C. While the sample in panel i shows no skew, indicating that noise both in the exponential sample and the stationary sample are comparable, the sample in panel ii shows a clear skew of all noisy data points above the level of neighbouring data points, while the sample in panel iii shows a skew below the level of neighbouring data points. We

do not currently know what is causing these variations even though we have run extensive tests to try to identify their cause. From our tests we suspect that a combination of factors including quality of genomic DNA preparation and library generation contributes to this effect. Whatever the reason, these problems affect mostly the noise and do not obscure the general trend of the bulk of the data points.

We have by now identified another effect that is specifically caused by the quality of the genomic DNA. The genomic DNA extraction via the GenElute Bacterial Genomic DNA Kit (Sigma-Aldrich) requires a 30 min proteolytic digest with proteinase K. This digestion step is not sufficient to fully remove all proteins in the sample. As a consequence, some partially digested or undigested proteins remain bound to DNA fragments. As part of the following column purification procedure these proteins including the DNA bound are removed. This causes areas of the chromosome in which proteins are tightly bound (*ter*/Tus complexes are one example) or which are very frequently bound by proteins (highly transcribed areas such as the *rrn* operons) to be under-represented in the genomic DNA preparation, leading to small dips in the profile. Examples can be seen in Figure 4. In Figure 4A panel i the clear peak at *oriC* appears to be missing in the *ΔrecG* sample. This absence of an *oriC* peak was not observed before (7, 18) and an analysis of the doubling time showed that *ΔrecG* cells have only a very mild growth defect (19), demonstrating that the frequency of *oriC* firing is not affected by the absence of RecG. We believe that the absence of a clear peak is caused by depletion of *rrn* operons *C*, *A*, *B* and *E*, which are in close proximity to *oriC*. Indeed, a reduction of the *oriC* peak height is not observed in the profiles shown Figure 5A, where growth is much slower due to the use of M9 glucose medium.

SUPPLEMENTARY FIGURE LEGENDS

Suppl. Figure 1. Over-replication in the termination area of *ΔrecG* cells proceeds through *terC* but not *terA*. The replication profiles for wild type and *ΔrecG* are reproduced from Figure 4A. The over-replication in the termination area in *ΔrecG* cells is enlarged to show at which *ter*/Tus complexes it is trapped. The number of reads (normalised against reads for a stationary phase wild type control) is plotted against the chromosomal location. A schematic representation of the *E. coli* chromosome showing positions of *oriC* and *ter* sites (above) as well as *dif* and *rrn* operons A–E, G and H (below) is shown above the plotted data. The strains used were MG1655 (wild type) and N4560 (*ΔrecG*).

Suppl. Figure 2. Impact of DNA polymerases II and V on over-replication in the termination area. Shown are spot dilution assays to evaluate the ability of origin-independent growth of *dnaA(ts) Δtus rpo* ΔrecG* cells in the presence or absence of DNA polymerases II and V. The strains used were RCe519 (*dnaA46 ΔrecG Δtus rpo**), JD1440 (*dnaA46 ΔrecG Δtus rpo* ΔpolB*) and JD1441 (*dnaA46 ΔrecG Δtus rpo* ΔumuCD*).

Suppl. Figure 3. Replication dynamics in *ΔrecG* and *ΔrecG Δtus* cells in strain backgrounds with one or two replication origins. **A)** Replisome numbers (YPet-DnaN) in the presence and absence of RecG helicase and a functional replication fork trap (*Δtus*). Shown are three individual experiments. 120 cells per strain and experiment were analysed. For *ΔrecG* and *ΔrecG Δtus* counts all filamentous cells with aberrantly increased foci numbers were excluded from the analysis. The strains used were AS1062 (*ypet-dnaN*), RCe766 (*ypet-dnaN ΔrecG*), RCe777 (*ypet-dnaN Δtus*) and RCe768 (*ypet-dnaN Δtus ΔrecG*). **B)** Replisome numbers (YPet-DnaN) in *oriC*$^{+}$ *oriZ*$^{+}$ cells in the presence and absence of RecG helicase and a functional replication fork trap (*Δtus*). The strains used were RCe749 (*oriC*$^{+}$ *oriZ*$^{+}$ *ypet-dnaN*), RCe773 (*oriC*$^{+}$ *oriZ*$^{+}$ *ypet-dnaN ΔrecG*), RCe758 (*oriC*$^{+}$ *oriZ*$^{+}$ *ypet-dnaN Δtus*) and RCe775 (*oriC*$^{+}$ *oriZ*$^{+}$ *ypet-dnaN Δtus ΔrecG*).

Suppl. Figure 4. Filamented cells in *ΔrecG* and *oriC*$^{+}$ *oriZ*$^{+}$ *ΔrecG* cells in the presence or absence of a functional replication fork trap (*Δtus*). Shown is the fraction of cells that showed a clear filamentation phenotype together with an aberrantly increased number of replisome (YPet-DnaN) foci. Cells were grown in M9 minimal salts media with glucose as described in Material and Methods. The number 0f filaments out of 400 cells per experiment were estimated. Figures plotted are the average from 3 independent experiments. Error bars indicate standard deviation.

Suppl. Figure 5. Comparative replication dynamics in wild type and *ΔrecG* cells grown either in LB broth or M9 minimal salts medium with glucose. The number of reads were normalised against reads for a stationary phase wild type control grown in the same medium and plotted against the chromosomal location. A schematic representation of the *E. coli* chromosome showing positions of *oriC* and *ter* sites (above) as well as *dif* and *rrn* operons A–E, G and H (below) is shown above the plotted data. The strains used were MG1655 (wild type) and N4560 (*ΔrecG*).

REFERENCES

1. Wang,X., Lesterlin,C., Reyes-Lamothe,R., Ball,G. and Sherratt,D.J. (2011) Replication and segregation of an *Escherichia coli* chromosome with two replication origins. *Proc. Natl. Acad. Sci. U. S. A.*, **108**, E243-250.

2. Woodgate,R. (1992) Construction of a *umuDC* operon substitution mutation in *Escherichia coli*. *Mutat. Res. Lett.*, **281**, 221–225.

3. Bachmann, B J (1996) Derivations and Genotypes of Some Mutant Derivatives of *Escherichia coli* K-12. In *Escherichia coli and Salmonella Cellular and Molecular Biology*. ASM Press.

4. Zhang,J., Mahdi,A.A., Briggs,G.S. and Lloyd,R.G. (2010) Promoting and avoiding recombination: contrasting activities of the *Escherichia coli* RuvABC Holliday junction resolvase and RecG DNA translocase. *Genetics*, **185**, 23–37.

5. Rudolph,C.J., Mahdi,A.A., Upton,A.L. and Lloyd,R.G. (2010) RecG protein and single-strand DNA exonucleases avoid cell lethality associated with PriA helicase activity in *Escherichia coli*. *Genetics*, **186**, 473–492.

6. Mahdi,A.A., Buckman,C., Harris,L. and Lloyd,R.G. (2006) Rep and PriA helicase activities prevent RecA from provoking unnecessary recombination during replication fork repair. *Genes Dev.*, **20**, 2135–2147.

7. Rudolph,C.J., Upton,A.L., Stockum,A., Nieduszynski,C.A. and Lloyd,R.G. (2013) Avoiding chromosome pathology when replication forks collide. *Nature*, **500**, 608–611.

8. Meddows,T.R., Savory,A.P. and Lloyd,R.G. (2004) RecG helicase promotes DNA double-strand break repair. *Mol. Microbiol.*, **52**, 119–132.

9. Jaktaji,R.P. and Lloyd,R.G. (2003) PriA supports two distinct pathways for replication restart in UV-irradiated *Escherichia coli* cells. *Mol. Microbiol.*, **47**, 1091–1100.

10. Guy,C.P., Atkinson,J., Gupta,M.K., Mahdi,A.A., Gwynn,E.J., Rudolph,C.J., Moon,P.B., van Knippenberg,I.C., Cadman,C.J., Dillingham,M.S., *et al.* (2009) Rep provides a second motor at the replisome to promote duplication of protein-bound DNA. *Mol. Cell*, **36**, 654–666.

11. Rudolph,C.J., Upton,A.L. and Lloyd,R.G. (2008) Maintaining replication fork integrity in UV-irradiated *Escherichia coli* cells. *DNA Repair*, **7**, 1589–1602.

12. Ivanova,D., Taylor,T., Smith,S.L., Dimude,J.U., Upton,A.L., Mehrjouy,M.M., Skovgaard,O., Sherratt,D.J., Retkute,R. and Rudolph,C.J. (2015) Shaping the landscape of the *Escherichia coli* chromosome: replication-transcription encounters in cells with an ectopic replication origin. *Nucleic Acids Res.*, **43**, 7865–7877.

13. Bernhardt,T.G. and de Boer,P.A.J. (2003) The *Escherichia coli* amidase AmiC is a periplasmic septal ring component exported via the twin-arginine transport pathway. *Mol. Microbiol.*, **48**, 1171–1182.

14. Datsenko,K.A. and Wanner,B.L. (2000) One-step inactivation of chromosomal genes in *Escherichia coli* K-12 using PCR products. *Proc. Natl. Acad. Sci. U. S. A.*, **97**, 6640–6645.

15. Upton,A.L., Grove,J.I., Mahdi,A.A., Briggs,G.S., Milner,D.S., Rudolph,C.J. and Lloyd,R.G. (2014) Cellular location and activity of *Escherichia coli* RecG proteins shed light on the function of its structurally unresolved C-terminus. *Nucleic Acids Res.*, **42**, 5702–5714.

16. Müller,C.A., Hawkins,M., Retkute,R., Malla,S., Wilson,R., Blythe,M.J., Nakato,R., Komata,M., Shirahige,K., de Moura,A.P.S., *et al.* (2014) The dynamics of genome replication using deep sequencing. *Nucleic Acids Res.*, **42**, e3.

17. Skovgaard,O., Bak,M., Løbner-Olesen,A. and Tommerup,N. (2011) Genome-wide detection of chromosomal rearrangements, indels, and mutations in circular chromosomes by short read sequencing. *Genome Res.*, **21**, 1388–1393.

18. Wendel,B.M., Courcelle,C.T. and Courcelle,J. (2014) Completion of DNA replication in *Escherichia coli.* *Proc. Natl. Acad. Sci. U. S. A.*, **111**, 16454–16459.

19. Rudolph,C.J., Upton,A.L., Harris,L. and Lloyd,R.G. (2009) Pathological replication in cells lacking RecG DNA translocase. *Mol. Microbiol.*, **73**, 352–366.

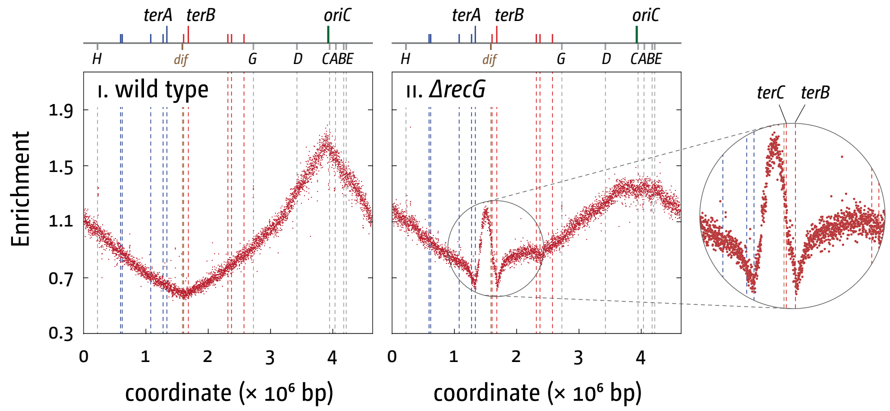

Midgley-Smith *et al.* Supplementary Figure 1

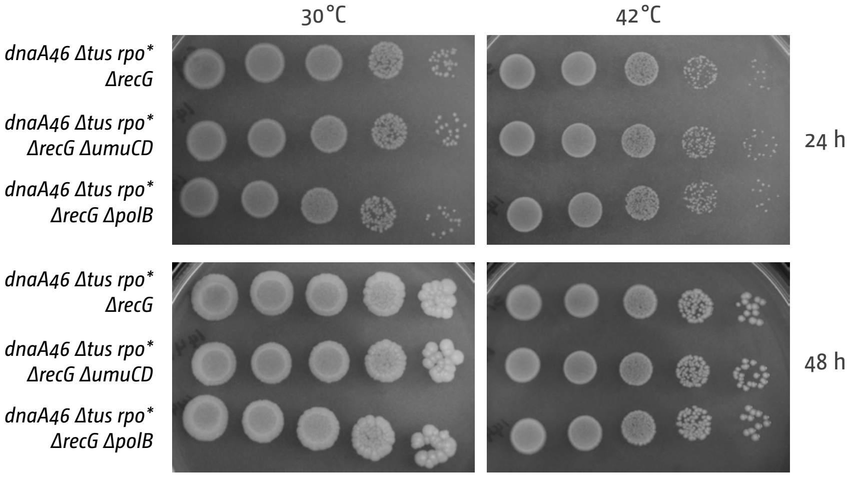

Midgley-Smith *et al.* Supplementary Figure 2

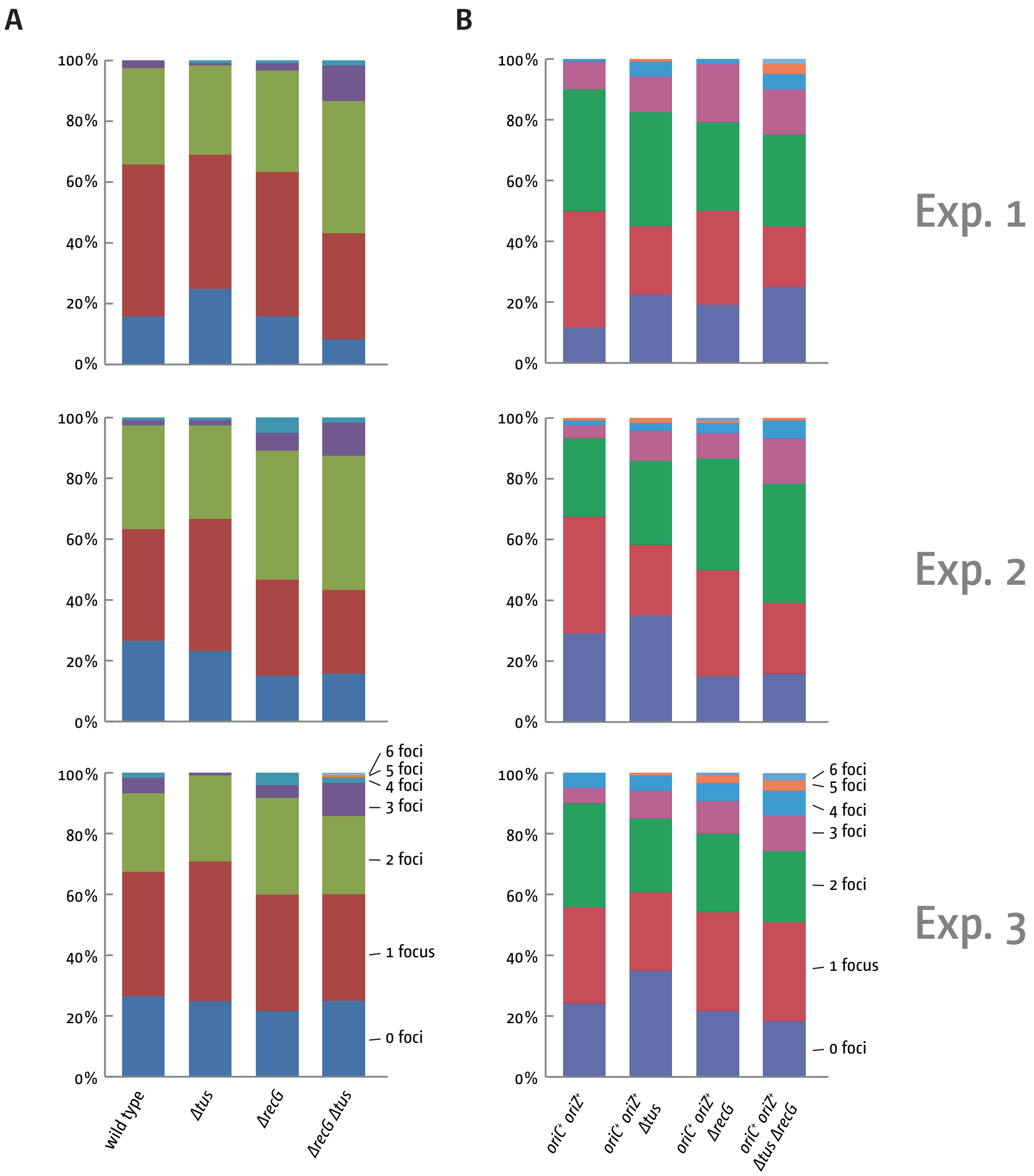

Midgley-Smith *et al.* Supplementary Figure 3

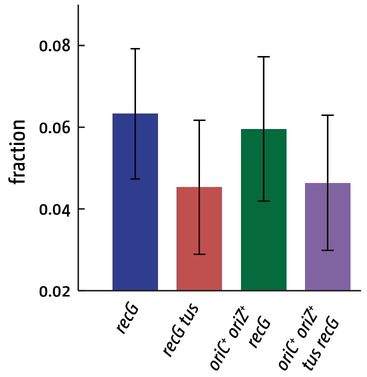

Midgley-Smith *et al.* Supplementary Figure 4

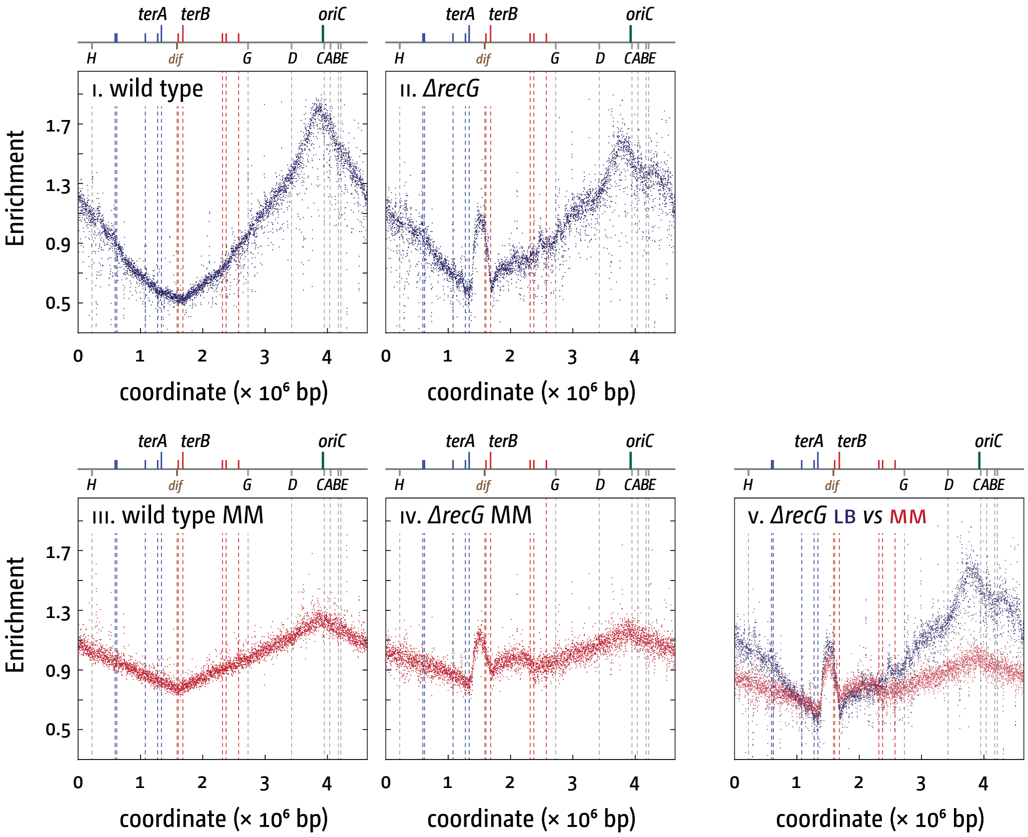

Midgley-Smith *et al.* Supplementary Figure 5
